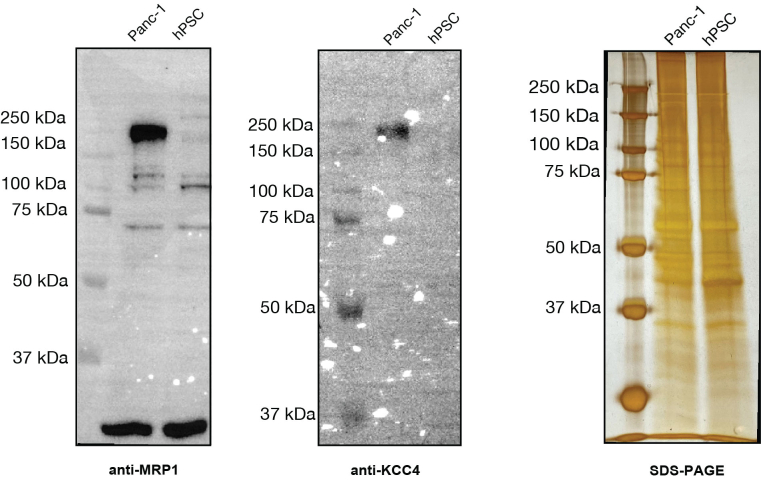# A Peptidisc-Based Survey of the Plasma Membrane Proteome of a Mammalian Cell

**DOI:** 10.1016/j.mcpro.2023.100588

**Published:** 2023-06-07

**Authors:** Zhiyu Zhao, Arshdeep Khurana, Frank Antony, John W. Young, Keeley G. Hewton, Zora Brough, Tianshuang Zhong, Seth J. Parker, Franck Duong van Hoa

**Affiliations:** 1 Department of Biochemistry and Molecular Biology, Faculty of Medicine, Life Sciences Institute, University of British Columbia, Vancouver, British Columbia, Canada; 2 British Columbia Children's Hospital Research Institute, Vancouver, British Columbia, Canada; 3 Centre for Molecular Medicine and Therapeutics, The University of British Columbia, Vancouver, British Columbia, Canada

**Keywords:** cell surface, transmembrane, membrane mimetic, lipids, transporters, channels, receptors, pancreas, cancer

## Abstract

Membrane proteins play critical roles at the cell surface and their misfunction is a hallmark of many human diseases. A precise evaluation of the plasma membrane proteome is therefore essential for cell biology and for discovering novel biomarkers and therapeutic targets. However, the low abundance of this proteome relative to soluble proteins makes it difficult to characterize, even with the most advanced proteomics technologies. Here, we apply the peptidisc membrane mimetic to purify the cell membrane proteome. Using the HeLa cell line as a reference, we capture 500 different integral membrane proteins, with half annotated to the plasma membrane. Notably, the peptidisc library is enriched with several ABC, SLC, GPCR, CD, and cell adhesion molecules that generally exist at low to very low copy numbers in the cell. We extend the method to compare two pancreatic cell lines, Panc-1 and hPSC. Here we observe a striking difference in the relative abundance of the cell surface cancer markers L1CAM, ANPEP, ITGB4, and CD70. We also identify two novel SLC transporters, SLC30A1 and SLC12A7, that are highly present in the Panc-1 cell only. The peptidisc library thus emerges as an effective way to survey and compare the membrane proteome of mammalian cells. Furthermore, since the method stabilizes membrane proteins in a water-soluble state, members of the library, here SLC12A7, can be specifically isolated.

## Highlights

• Peptidisc stabilizes membrane proteins in a water-soluble library.
• The membrane protein library is amenable to purification and proteomic analysis.
• The library comprehensively reflects the membrane proteome.
• The method facilitates the identification of cell surface markers.

## Graphical Abstract

**Figure:**
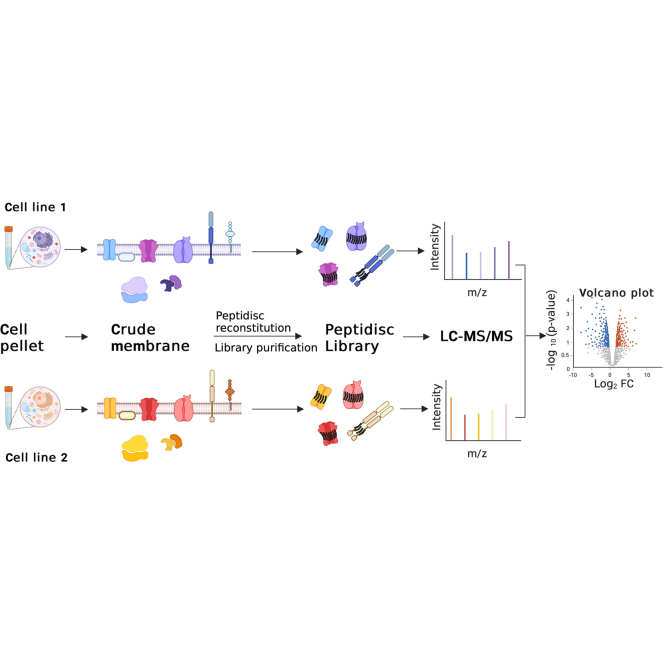

## In Brief

Membrane proteins are difficult to detect in bottom-up proteomics due to their hydrophobicity and low abundance compared to soluble proteins. We apply here the peptidisc membrane-mimetic to isolate the plasma membrane proteome. The method captures membrane proteins in a water-soluble state that is amenable to mass spectrometry analysis and protein purification. The technique is promising in identifying naturally low abundant plasma membrane proteins. It is also applicable to compare the panels of cell surface markers.

Membrane proteins (MPs), especially plasma MPs, play critical roles in cellular communication and interactions with their surroundings. They fulfill numerous functions, such as signal transduction, nutrient transport, cell adhesion, antigen presentation, drug extrusion, and many other enzymatic activities (1, 2, 3). Misfunction, mistargeting, or altered expression of MPs are directly linked to disease development, including hypertension (4), Alzheimer’s disease (5), and multiple forms of cancers (6, 7, 8, 9). Due to their central involvement in disease development and surface accessibility, MPs—particularly G protein-coupled receptors (GPCRs), solute carriers, and ion channels—are among the most amenable and lucrative targets for diagnostic and therapeutic research (10, 11, 12, 13).

Mass spectrometry (MS) is a very valuable method to survey a cell integral membrane proteome, yet challenging to perform because MPs are poorly soluble and present in low abundance compared to the overall cell proteome (14, 15). During MS analysis, the intense signals derived from soluble proteins often mask the weaker signal obtained with integral membrane proteins (IMPs, (10, 16, 17, 18). A few methods have been developed to increase the detection efficiency of this membrane proteome, such as silica-bead coating, cell surface labeling (CSL), and cell surface capture (CSC) (19, 20, 21, 22). These methods have greatly improved the definition of the membrane proteome, yet some technical challenges remain. The CSL procedure is limited by the surface accessibility of lysine residues, and over-labeling and cell lysis can decrease digestion efficiency and labeling specificity, respectively (23, 24). The CSC method also depends on the selective capture of a glycosyl-moiety that can be heterologous and absent on certain cell surface proteins (23, 24). The recovery of the membrane proteome is also problematic because membrane solubilization often requires detergents, such as SDS, which are not compatible with the downstream LC-MS (24, 25, 26). Protocols, such as filter-aided sample preparation and S-Trap, have been developed to help with detergent removal, but it is still challenging to recover peptides without causing loss (27, 28, 29, 30, 31).

In this study, we test if the membrane mimetic peptidisc, so far developed with *Escherichia coli*, can also be employed to survey the lipid-diverse and organelle-complex mammalian membrane proteome. And if yes, how do the results compare to other cell surface assays such as CSC and CSL? The peptidisc sensitive enough to perform comparative membrane analysis without requiring labeling reagents or strong detergents. The peptidisc is an amphipathic ApoA1-derived peptide designed to shield the water-insoluble parts of MPs (18, 32, 33, 34). Due to a self-assembly property, this mimetic can convert the whole membrane proteome into water-soluble nanoparticles, termed as “peptidisc library” (32, 35). Importantly, for this study, the peptidisc is modified with a His_6_-tag, and thus the membrane proteome in the library can be enriched by nickel nitrilotriacetic acid (Ni-NTA) chromatography (18). Since the library bypasses strong detergents, the protein folds are preserved and proteins can be purified.

Using the HeLa cell line as a reference, we report the specific captures of ∼500 IMPs, with half predicted to be located at the plasma membrane (pIMPs). As expected, the IMPs representation over total proteins is greatly augmented upon library purification. These identification results compare well with other CSL and CSC methods. We then apply the method to survey the membrane proteome of the pancreatic ductal adenocarcinoma (PDAC) cell line Panc-1 and the nonmalignant stellate cell human pancreatic stellate cell (hPSC) (36). PDAC is one of the most lethal cancers, partly due to a lack of effective therapeutics (37, 38). Our results provide a short list of plasma MPs, whose abundance is strikingly different across the two cell lines, including known and potentially novel biomarkers and actionable targets. We further show that one of them can readily be isolated from the library using an antibody.

## Experimental Procedures

### Materials

Frozen HeLa cell pellets (C3 PN: HA48) were purchased from the Cell Culture Company. Nickel-affinity resin was obtained from Qiagen. Detergent n-dodecyl-β-D-maltoside (DDM) was purchased from Anatrace. His-tagged peptidiscs (purity >90%) were obtained from Peptidisc Biotech. Superose 6 10/300 and Protein A Sepharose CL-4B were purchased from GE Healthcare. The protease inhibitor cOmplete Cocktail was purchased from Sigma. Trypsin, anti-KCC4 (A304-442A), and anti-MRP1 (A304-419A) antibodies were purchased from Thermo Fisher Scientific. Octadecyl (C18) Empore disks were purchased from 3M. Polygoprep 300-20 C18 power was purchased from Macherey-Nagel. General chemicals such as NaCl, Tris-base, PMSF, and EDTA were obtained from Bioshop and Thermo Fisher Scientific Canada. Anti-Na^+^/K^+^ ATPase (sc-21712) was kindly provided by Santa Cruz Biotechnology for a sample test.

### Cell Cultures

The cell line Panc-1 was obtained from the American Type Culture Collection, a nonprofit organization for cell line collection, and authenticated by short tandem repeat DNA fingerprinting and maintained in a centralized cell bank. The de-identified hPSC#1 cell line was generously supplied under a transfer agreement from Alec Kimmelman at the New York University School of Medicine (36). Both cell lines were verified to be negative for *Mycoplasma* by PCR method prior to experiments. Cell lines were cultured in Dulbecco’s modified Eagle’s medium (Corning) supplemented with 10% fetal bovine serum and 1% penicillin-streptomycin. Roughly 40 million cells were harvested per sample by rapid trypsinization and washed twice in PBS. Cell pellets were flash-frozen in liquid nitrogen and stored at −80 °C until use.

### Preparation of Crude Membranes

Frozen cells pellets (equivalent to ∼40 million cells) were resuspended in 4 ml hypotonic buffer (10 mM Tris–HCl, 30 mM NaCl, and 1 mM EDTA, pH 7.4) containing 1× cocktail protease inhibitor and 1 mM PMSF on ice for 20 min. Cells were homogenized in a metal douncer and through a 27 Gauge needle over 70 times. To remove chromosomal DNA, 10 mM MgCl_2_ and 50 μg Dnase were added to the lysed cells and incubated on ice for 15 min. Unbroken cells and nucleus fraction were removed by centrifugation at 1200*g* for 10 min at 4 °C. The supernatant was collected and centrifuged again (5000*g*, 10 min at 4 °C) to remove the mitochondria fraction. The crude membrane fraction (plasma, endoplasmic reticulum [ER], Golgi, vesicle membranes) was then pelleted by ultra-centrifugation (110,000*g*, 45 min at 4 °C) in a Beckman TLA110 rotor. This membrane preparation was resuspended in 100 μl TSG buffer (50 mM Tris, pH 7.8, 100 mM NaCl, 10% glycerol) and stored at −80 °C until use.

### Preparation of the Peptidisc Libraries

To prepare the peptidisc library, crude membranes were solubilized in 0.8% DDM for 30 min at 4 °C with gentle shaking. After the removal of insoluble aggregates by ultracentrifugation (100,000*g*, 15 min, 4 °C), the MPs present in the detergent extract was reconstituted into His-tagged peptidiscs as previously described with minor modifications (18). Briefly, the “DDM extract” (∼300 μg) was mixed with His-tagged peptidisc peptide (∼900 μg) for 15 min at 4 °C. The mixture (total ∼450 μl) was diluted to 5 ml in TS buffer (50 mM Tris, pH 7.8, 100 mM NaCl) over a 100 kDa-cutoff centrifugal filter (Amicon, Millipore) and then concentrated (3000*g*, 10 min). The mixture (∼200 μl) was diluted to 5 ml again in TS buffer and concentrated to ∼300 μl (∼1 mg). The resulting peptidisc library (termed “Starting Library”) was incubated with 60 μl of Ni-NTA resin (Qiagen) for 1 h with shaking. After extensive washing with TS buffer to remove nonspecific binders (5 washes, 1 ml each), the “Purified Library” was eluted in 150 μl TS buffer supplemented with 600 mM imidazole.

### Detergent Removal and Acetone Precipitation

Sample “DDM Extract” preparation: The DDM-solubilized crude membrane (180 μg) was treated with 100% ice-cold acetone and left overnight at −20 °C to precipitate. The precipitated proteins were pellet at 16,100*g* for 10 min. The pellet was then washed with 100% acetone and pelleted again. The supernatant was aspirated, and the pellet was air-dried at 42 °C. The pellet was resuspended in 100 μl of 20 mM NH_4_HCO_3_ before trypsin digestion.

### Immunoprecipitation of KCC4

About 4 μg of anti-KCC4 antibody were incubated with Panc-1 peptidisc library (∼1 mg) in TS buffer (50 mM Tris, 100 mM NaCl) overnight at 4 °C with gentle rocking. On the following day, the mixture was incubated with 200 μl Protein A Sepharose resin equilibrated with TS buffer for at least 4 h with gentle rocking. After two washes with TS buffer (1 ml and 1 min each), the immunoprecipitation (IP) sample was eluted with 200 μl of 100 mM glycine (pH 3.0). Before 6 M urea treatment and trypsin digestion, the pH of the IP sample was adjusted to 7.8 using 1 M Tris.

### Sample Preparation for MS Analysis

Protein samples (DDM Extract, Starting Library, Purified Library, ∼80 μg each; IP sample, concentration not measurable) were treated with 6 M urea at room temperature for 30 min before reduction with 10 mM fresh DTT for 1 h. Alkylation was performed with 20 mM iodoacetamide in the dark at room temperature for 30 min, followed by the addition of 10 mM DTT for 30 min. The urea concentration was diluted to 1 M with TS buffer. Trypsin digestion was performed with an enzyme/protein ratio of 1:50 at 25 °C for 18 h. The tryptic peptides were acidified to pH 3 with 10% formic acid and desalted using home-packed Stage-Tips C18. The peptides were eluted with 80% acetonitrile/0.1% formic acid and dried by vacuum centrifugation.

### LC and MS/MS Analysis

The LC and MS analysis was done at the SPARC BioCenter (The SickKids Proteomics). The dried peptides were resuspended in buffer A (0.1% formic acid). Peptides (3 μg for DDM-treated and 4 μg for the peptidisc library samples) were analyzed using an EASY-nanoLC 1200 system coupled to an Orbitrap Fusion Lumos Tribrid Mass Spectrometer (Thermo Fisher Scientific). The LC portion of the analysis consisted of an 18 min linear gradient running 3 to 20% of buffer A to buffer B (0.1% FA, 80% acetonitrile), followed by a 31 min linear gradient running 20 to 35% of buffer A to buffer B, a 2 min ramp to 100% buffer B and 9 min hold at 100% buffer B, all at a flow rate of 250 nl/min. Samples were loaded into a 75 μm × 2 cm Acclaim PepMap 100 Pre-column followed by a 75 μm × 50 cm PepMax RSLC EASY-Spray analytical column filled with 2 μm C_18_ beads (Thermo Fisher Scientific). MS1 acquisition resolution was set to 120,000 with automatic gain control target value of 4 × 10^5^ and maximum ion injection time of 50 ms for a scan range of *m/z* 375 to 1500. Monoisotopic precursor selection was determined at the peptide level with a global intensity threshold of 10,000. Only peptides with charge states of 2 to 7 were accepted, with dynamic exclusion set to 10 s. Isolation for MS2 scans was performed in the quadrupole with an isolation window of *m/z* 0.7. MS2 scans were performed in the ion trap with a maximum ion injection time of 10 ms, automatic gain control target value of 1 × 10^4^, and higher-energy collisional dissociation activation with a normalized collision energy of 30.

### Raw Data Processing

All raw MS data were processed using MaxQuant (https://www.maxquant.org) v2.0.3.0 (39). MaxQuant-integrated Andromeda search engine was used against the UniProt *Homo sapiens* (modified seventh of March, 2021—78,120 entries, unreviewed) protein database. To allow Andromeda to recognize the peptidisc peptides in the samples, the NSPr sequence was manually added to the protein database and given the arbitrary protein ID, P1EPTD (NSPr sequence: n-FAEKFKEAVKDYFAKFWD-P-AAEKLKEAVKDYFAKLWD-c). The initial MaxQuant individual peptide mass tolerance was set at 20 ppm for both the precursor and fragment ions. The entries in the database were trypsin digested *in silico* and matched against detected peptide features, with a maximum of two missed cleavages considered. Cysteine carbamidomethyl was set as a fixed modification, while methionine oxidation, N-terminal acetylation, asparagine, and glutamine deamidation were the variable modifications. The UniProt database was also concatenated with an automatically generated reverse database to estimate the false discovery rate using a target decoy search. A threshold false discovery rate of 1% was applied at the peptide spectrum match and protein level. For relative quantification, the MaxQuant label-free quantification function—label-free quantitation (LFQ) and iBAQ—were enabled (40). Both razor and unique peptides were used for quantification.

### Statistical Analysis

The protein groups.txt output from MaxQuant was exported into Perseus v1.6.15.0 for downstream analysis (41). The protein groups identified from the reverse decoy database, marked as potential contaminants, or only identified by a posttranslation modification site were removed from any downstream analysis. The remaining intensity, LFQ intensity, and iBAQ values were log2 normalized. For the HeLa, Panc-1, and hPSC Purified Library replicates, an LFQ analysis was performed to assess the reproducibility with a Pearson correlation coefficient. To find the differential abundance of proteins in the Panc-1 and hPSC cell line libraries, a student’s *t* test was conducted with an artificial within groups variance, s0, set at 0.1. The test was applied on data filtered for only those proteins with a valid LFQ intensity in at least both replicates of the hPSC or Panc-1 cell line libraries. Before applying the *t* test, the remaining undefined intensity values were imputed from a normal distribution with a downshift of 1.8 SDs from the total sample mean and a width of 0.3 times the sample SD. In this study, proteins are considered differentially expressed between the cells if the peptide intensity (PI) fold change (FC) across samples is ≥2 or ≤-2 (the absolute value of Log_2_FC ≥ 1), *p* < 0.05 (-Log_10_ (*p*-value) > 1.3).

### Protein Annotation

The protein list was “Gene ontology (GO)-term” analyzed using the UniProtKB database. Proteins with the GO-term “membrane” were annotated as MPs. The Phobius web server (http://phobius.sbc.su.se/) was then used to predict the number of the transmembrane segment (TMS) for each MP. Proteins with at least one predicted TMS were annotated as IMPs. The subcellular location of the IMPs was then categorized using the GO-term “Subcellular location [CC]” to “plasma membrane,” “ER/Golgi membrane,” “vesicle membrane (including endosome, exosome, lysosome, vesicle, and peroxisome),” “mitochondrial membrane,” and other “membrane (including “membrane,” nucleus, and secreted)” proteins. The IMPs with a GO-term “plasma membrane” or “cell membrane” were annotated as pIMPs. The IMPs that were solely defined with a GO-term “membrane” were further analyzed using the HeLa spatial proteome database to get their specific localization (42).

### Other Bioinformatic Tools

Venn Diagram analysis was done using Bioinformatics & Evolutionary Genomics (http://bioinformatics.psb.ugent.be/webtools). The relative abundance of MPs was checked in PAXdb: Protein Abundance Database (https://www.pax-db.org) using the gene name of each protein. Graphs and corresponding statistical analysis were done in GraphPad Prism 8.4.3 (https://www.graphpad.com). The Graphic Abstract was created with BioRender (https://biorender.com).

### Western Blot

Crude membranes (5 μg) from either Panc-1 or hPSC were loaded on 10% SDS-PAGE. The proteins were transferred to a polyvinylidene fluoride membrane (Immobilon-P, Millipore) *via* semi-dry Western Blotting. Anti-KCC4 (SLC12A7), anti-MRP1 (ABCC1), or anti-Na^+^/K^+^ ATPase (ATP1A1) were used as primary antibodies and incubated with the membrane overnight at 4 °C. After washing with PBS-Tween (3 times, 10 min each), the HRP-conjugated secondary antibody (anti-rabbit or anti-mouse) was added to the membrane for 1 h at room temperature. The membrane was washed with PBS-Tween (6 times, 5 min each). The chemiluminescent substrate (170-5060, Bio-Rad) was incubated with the membrane for 5 min before imaging.

### Experimental Design and Statistical Rationale

Proteomic studies on Panc-1 and hPSC cells were conducted in biological triplicates (n = 3). The MS data analysis was done using MaxQuant and Perseus. To investigate the reproducibility of the peptidisc library purification method, we used the MaxLFQ algorithm for LFQ. Quantification was performed using both razor and unique peptides. The correlation coefficient r was obtained using Perseus. For the comparison between Panc-1 and hPSC cells, a student’s *t* test was performed. Proteins were considered differentially expressed when the FC of their PI across the two samples was equal to or larger than 2, and a *p*-value <0.05 was considered statistically significant.

## Results

### Capture and Purification of the HeLa Cell Membrane Proteome in Peptidisc

The HeLa cell line is commonly employed in proteomics analysis (43, 44, 45), and its cell surface proteome was profiled using the CSL and CSC methods (46, 47). We, therefore, employed this cell line to benchmark our study. After mechanical lysis of ∼40 million cells, the membrane fraction was isolated by ultracentrifugation to remove nuclei and large organelles such as mitochondria. Next, the membrane fraction, a mixture of plasma, ER, and Golgi membranes, was solubilized with the detergent DDM (hereafter termed “DDM Extract”). The proteins in the DDM Extract were then trapped in the His-tagged peptidisc (termed “Starting Library”) and purified over Nickel-affinity resin (termed “Purified Library”). Following these preparative steps, equal amounts of DDM Extract, Starting Library, and Purified Library (∼80 μg protein each) were digested with trypsin, stage-tipped, and analyzed by LC-MS/MS. The proteins identified were then annotated using the GO-term “membrane” to determine the number of MPs. Within the MPs group, proteins containing at least one α-helical TMS were labeled IMPs. The IMPs with the GO-term “plasma membrane” or “cell membrane” were further annotated as pIMPs.

The DDM Extract contained 2646 protein IDs, compared to 2186 in the Starting Library. We note that similar amounts of digested peptides were analyzed in both cases (3–4 μg), but since 3/4th of the peptide mass comes from the peptidisc peptides in the latter case, a lower identification rate is expected. We also note that ∼300 fewer proteins were identified in the Purified Library than the Starting Library (1865 vs. 2186; Table 1), but the protein annotation reveals that most of the missed IDs (∼90%) correspond to cytosolic proteins (*e.g.*, translational, proteasomal, and RNA-related proteins), as expected since these nonmembrane contaminants are washed away during library purification. Comparing the three samples, the percentage of IMPs and pIMPs identified over the total protein IDs was similar (∼23–29% for IMPs and 8–14% for pIMPs; Table 1). However, as shown below, the detection efficiency of IMPs is greatly augmented in the Purified Library.

**Table 1:** Number of proteins identified across the various samples analyzed

MS sample	Total proteins	MPs	IMPs	pIMPs	Ratio IMPs/Total proteins	Ratio pIMPs/Total proteins
DDM extract	2646	992	601	226	27.7%	8.5%
Starting library	2186	912	509	235	23.3%	10.8%
Purified library	1865	877	534	249	28.6%	13.4%
Reference list 1 (Li *et al.*, 2019) (46)	1899	854	528	258	27.8%	13.6%
Reference list 2 (Bausch-Fluck *et al.*, 2015) (47)	347	311	286	207	82.4%	59.6%

“Total proteins” represents the total number of proteins identified in each sample. “MPs” represents the proteins with a “membrane” GO-term. “IMPs” represents the proteins with at least one predicted transmembrane segment. “pIMPs” represents IMPs that have a “cell membrane” or “plasma membrane” GO-term. The Reference Lists (“supplemental Table S2_experimental group #1” in (46) and “supplemental File S1” HeLa dataset in (47)) were annotated following the same criteria. The complete annotated reference datasets can be found in supplemental File S1.

### Enrichment of pIMPs in the Purified Library

**Fig. 1:**
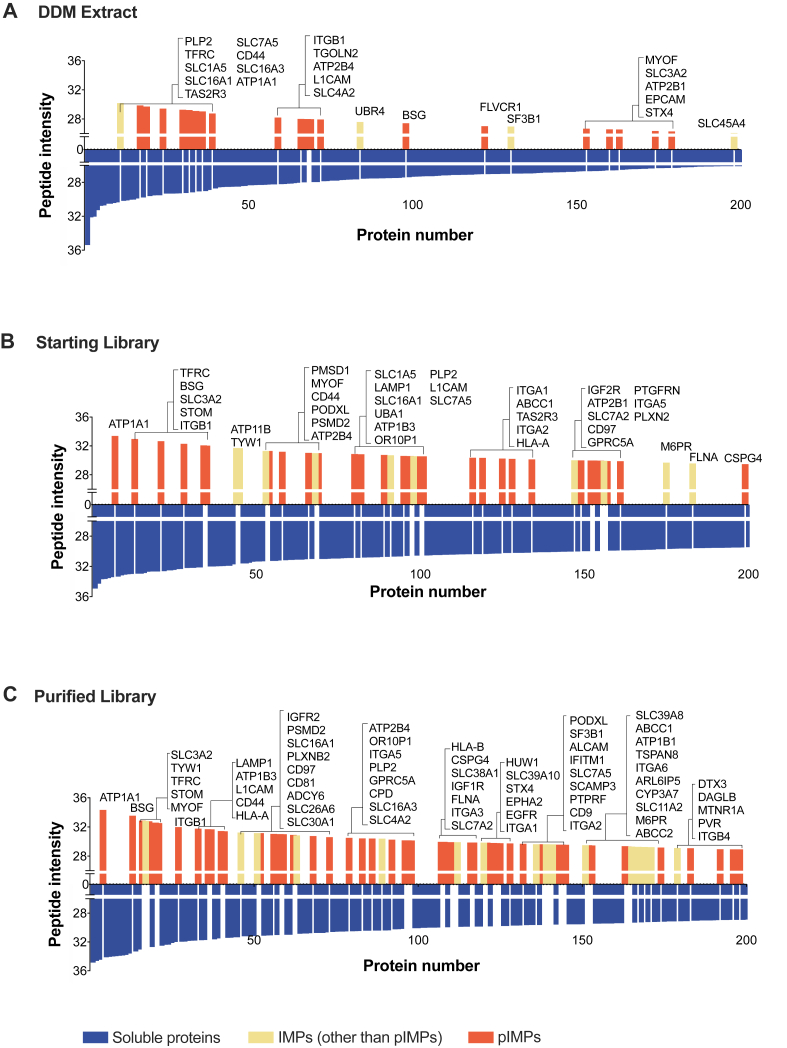
**Ranking of the top 200 identified proteins based on their peptide intensity.** Proteins identified in DDM extract (*A*), Starting Library (*B*), and Purified Library (*C*) are shown. The peptide intensity values are reported in supplemental File S1. Proteins without a predicted TMS are annotated as “Soluble proteins” and colored in *blue*, pIMPs are colored in *orange*, and the remaining IMPs (different than pIMPs) are colored in *light yellow*. The gene name of each (p)IMP is indicated in the figure. DDM, n-dodecyl-β-D-maltoside; IMP, integral membrane protein; pIMP, plasma integral membrane protein; TMS, transmembrane segment.

**Fig. 2:**
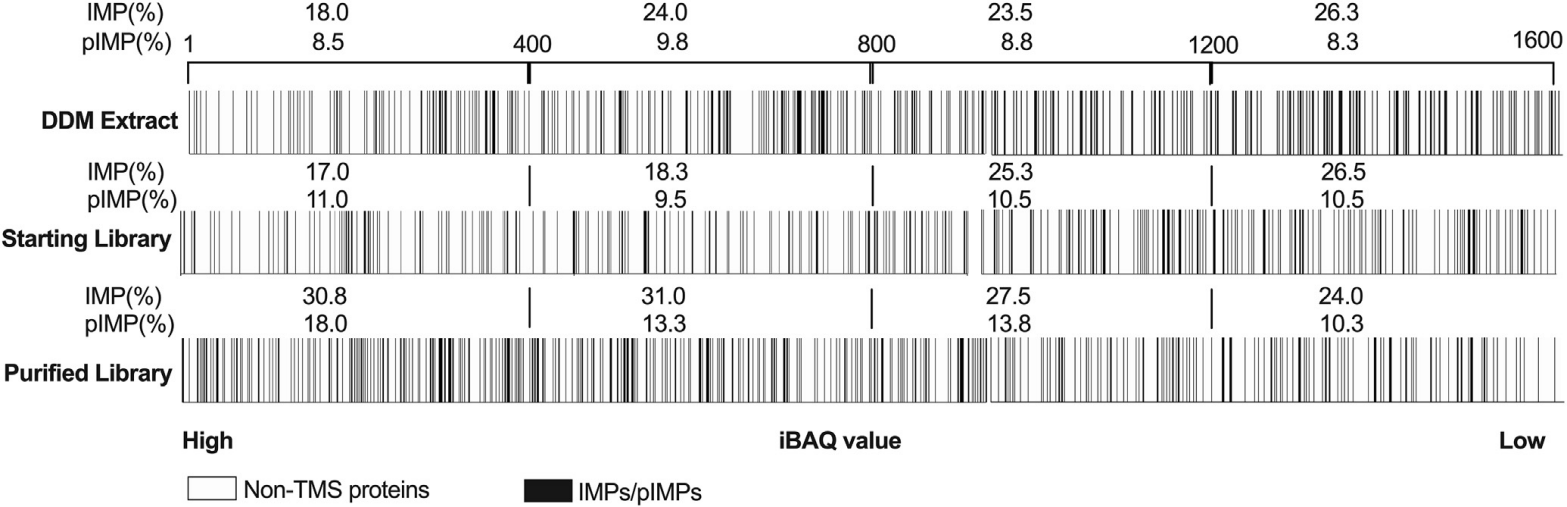
**Comparison of protein abundance within samples.** Proteins identified in DDM Extract, Starting Library, and Purified Library were ranked based on their iBAQ values. The top 1600 proteins from each sample are plotted and divided into quartiles. The presence of IMPs/pIMPs is provided as a % of the total proteins present in each quartile. DDM, n-dodecyl-β-D-maltoside; IMP, integral membrane protein; pIMP, plasma integral membrane protein.

To assess the impact of library purification, we ranked the proteins identified using their PI and iBAQ values, which reflect protein abundance across and within samples. To visualize the results, we plotted and annotated the top 200 proteins (Fig. 1 and supplemental File S1). The Purified Library had almost three-fold more IMPs than the DDM Extract (68 IMPs/52 pIMPs *versus* 24 IMPs/20 pIMPs, respectively) and almost twice more than in the Starting Library (compare Figure 1*B* to Figure 1*C*). Notably, some pIMPs considered key therapeutic targets, such as the solute carrier (SLC) transporters (SLC26A6, SLC30A1, SLC16A3, SLC4A2, SLC38A1, SLC39A10), integrins (ITGA3, ITGA6, ITGB4), and CD antigens (CD81, CD9), appeared in the top 200 IDs in the Purified Library only (Fig. 1*C*). We next plotted and compared the iBAQ values for the total proteins identified. As shown in Figure 2, the top 400 most abundant proteins contained twice more IMPs and pIMPs in the Purified Library than the corresponding sample from the Starting Library or DDM Extract. Altogether, these results show that library purification removes soluble background protein contaminants, which augments the detection efficiency of IMPs.

### Presence of Low-Abundance pIMPs in the Peptidisc Library

To assess the overall sensitivity of our method, we determined if the Purified Library contains pIMPs that otherwise exist in limited copy numbers in the HeLa cell. For this analysis, we used the Protein Abundance Database (PAXdb) (https://www.pax-db.org) because this reference database integrates the HeLa cell data obtained from three independent high-throughput proteomics studies, which provides maximal coverage of the HeLa proteome (48, 49, 50). Strikingly, over the 249 pIMPs we examined, 37 were either completely absent or in extremely low abundance in the PAXdb database (bottom 10%). The list includes the GPCRs (OR10P1, RXFP1, EMR2, OR14A16, GRM3, TAS2R3), SLC transporters (SLC13A4, SLC46A1, SLC19A3, and SLC22A1), membrane enzymes (CA9 and MMP15), and an ion channel (ORAI1). Other medically important pIMPs, such as ABC transporters (ABCC2, ABCC3), SLC (SLC7A2, SLC26A6, SLC39A8), and cell adhesion molecule (EPCAM), which rank bottom 50% in the database, were now listed at the top 25% in the Purified Library (supplemental File S2).

### Content of the HeLa Cell Peptidisc Library

**Fig. 3:**
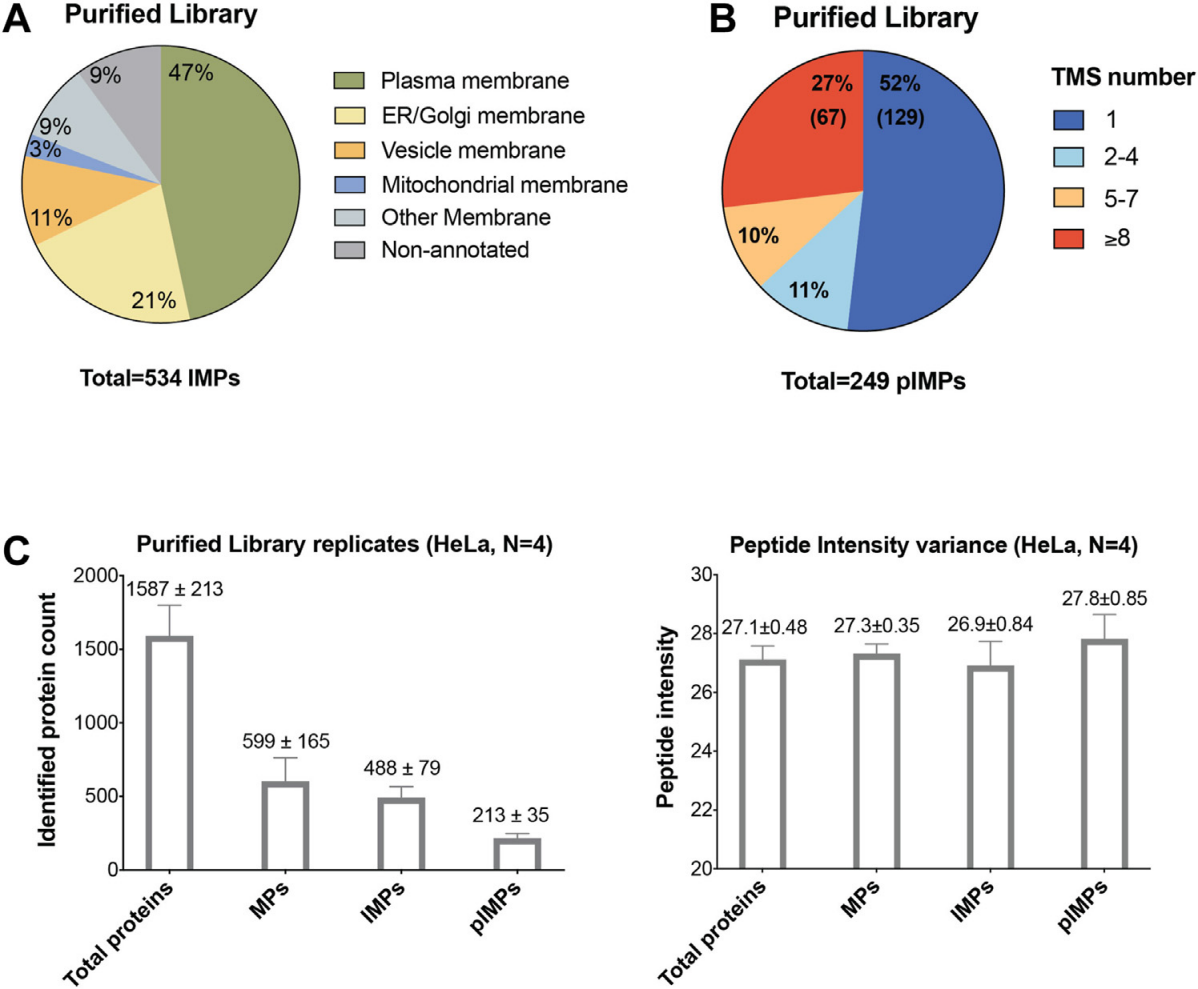
**Purified Library.***A*, subcellular location for the IMPs identified in the Purified Library. The annotation is based on the GO-term “Subcellular location [CC]” terminology. *B*, TMS number of identified pIMPs. The TMS number of each identified pIMP was annotated based on Phobius prediction. *C*, biological replicates. Variance on protein counts and peptide intensities obtained with the HeLa cell Purified Libraries. The numbers in the graph indicate the mean ± SD, N = 4. IMP, integral membrane protein; pIMP, plasma integral membrane protein; TMS, transmembrane segment.

The IMPs in the Purified Library were classified using their GO-term “Subcellular Location.” About half of the IMPs were assigned to the plasma membrane (249 pIMPs out of 534 IMPs). The remaining IMPs were assigned mainly to the ER/Golgi system and small vesicular membranes (endosome, exosome, lysosome; Fig. 3*A*). We also classified the 249 pIMPs using their GO-term “molecular function” (Table 2 and supplemental File S3). The classification indicates that over half of the pIMPs are functionally related to membrane transport (77 IDs, including SLC, ATPase pumps, ABC transporters), membrane enzymes (39 IDs, including kinases, peptidases, and phosphatases), and transmembrane receptors (70 IDs, including 13 GPCRs). The other pIMPs correspond to ion channels (8 IDs), adhesion molecules (16 IDs), CD antigens (9 IDs), regulators (14 IDs), chaperones (6 IDs), and vesicle-associated MPs (8 IDs), in addition to pIMPs (48 IDs) with unknown or less evident function.

**Table 2:** Molecular function of IMPs and pIMPs identified in Purified Library

Molecular function	Subtype	IMPs	pIMPs
Adaptor/chaperone/chaperone binding	−	12	6
Adhesion molecule	−	16	16
Enzyme	Kinase	15	12
	Peptidase	19	8
	Phosphatase	10	8
	Other	63	11
Membrane transport protein	ABC transporter	8	8
	ATPase pump	17	9
	SLC transporter	69	53
	Ion channel	18	8
	Other	27	7
Receptor	GPCRs	15	13
	Other	83	57
Regulator	−	30	14
Vesicle/Cargo protein	−	29	8
CD antigen	−	11	9
Other	−	147	48

The protein molecular function was retrieved from the UniprotKB “Gene Ontology (molecular function)”. Key words “adaptor activity”, “chaperone binding”, “chaperone”; “focal adhesion”; “receptor activity”; “regulator activity”; “G protein-coupled receptor activity”; “channel”; and “transporter” were used to look for the specific function of each IMP and pIMP. The molecular function annotation of ABC-transporter, ATPase pump, SLC transporter, and CD antigens were determined based on their gene name. The assigned molecular function of each IMP and pIMP is presented in supplemental File S3. Note that proteins can be classified with two or more molecular functions.

**Figure S1:**
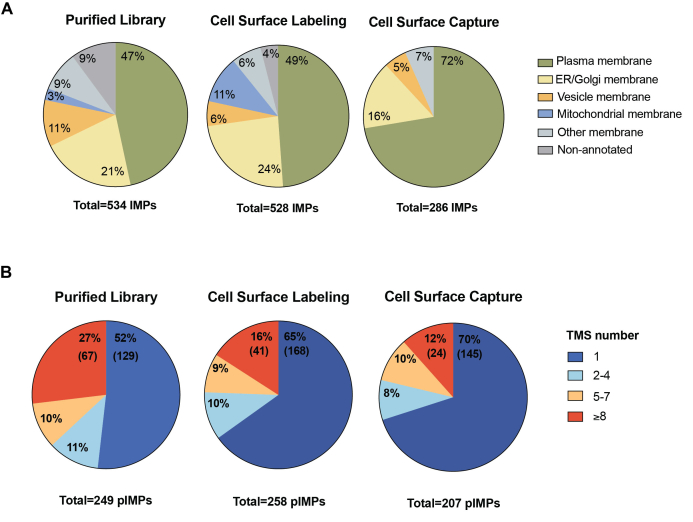


To further analyze our results, we compared our data to that published by Li *et al.* (46) and Bausch-Fluck *et al.* (47), which employed the CSL and CSC protocols to survey the HeLa membrane proteome. We downloaded the corresponding datasets and annotated them following the same criteria as in our study (supplemental Fig. S1*A* and supplemental File S1). As summarized in Table 1, the CSC method showed the best spatial resolution, since ∼60% of the total protein identified are classified as pIMPs, compared to ∼13 to 14% with the Peptidisc and CSL methods. However, Table 1 also shows that all three methods identified a similar number of pIMPs (∼210–250 IDs). Thus, the identification efficiency of pIMPs is seemingly identical for all three methods.

We also ranked the pIMPs based on their predicted TMS number (Figs. 3*B* and S1*B*). The comparison reveals that the CSL and CSC methods identify a higher number of pIMPs with one TMS (65%-70%, compared to 52% in peptidiscs). In contrast, the number of pIMPs with multiple TMS is seemingly higher with the peptidisc method (48% in peptidiscs vs. 30–35% in CSL/CSC; supplemental Fig. S1*B*).

Finally, to examine the variability of the method, we performed a biological quadruplicate (Fig. 3*C*). On average, 1600 proteins were detected in each replicate, with about 490 proteins annotated as IMPs and 210 as pIMPs. We obtained a generally good correlation between replicates (average r = 0.61, *p* < 0.0001, supplemental Fig. S2*A*), considering the high number of variables caused by cell lysis, centrifugation steps, and detergent solubilization, library construction, purification, and LC-MS/MS analysis.

### Comparing the Plasma Membrane Proteome of Two Pancreatic Cell Lines

Given the encouraging results, we tested our method in comparative proteomics. We used the Panc-1 cell line, commonly used to study PDAC *in vitro* (51), and the hPSC cell line, which is an immortalized but nonmalignant cell found in the PDAC microenvironment (38, 52, 53). The stellate cells supply the PDAC cells with metabolic fuels through the selective engagement of cell surface transporters (38, 54). Thus, the global proteomic comparison of the membrane systems in the two cell lines may provide valuable information.

**Fig. 4:**
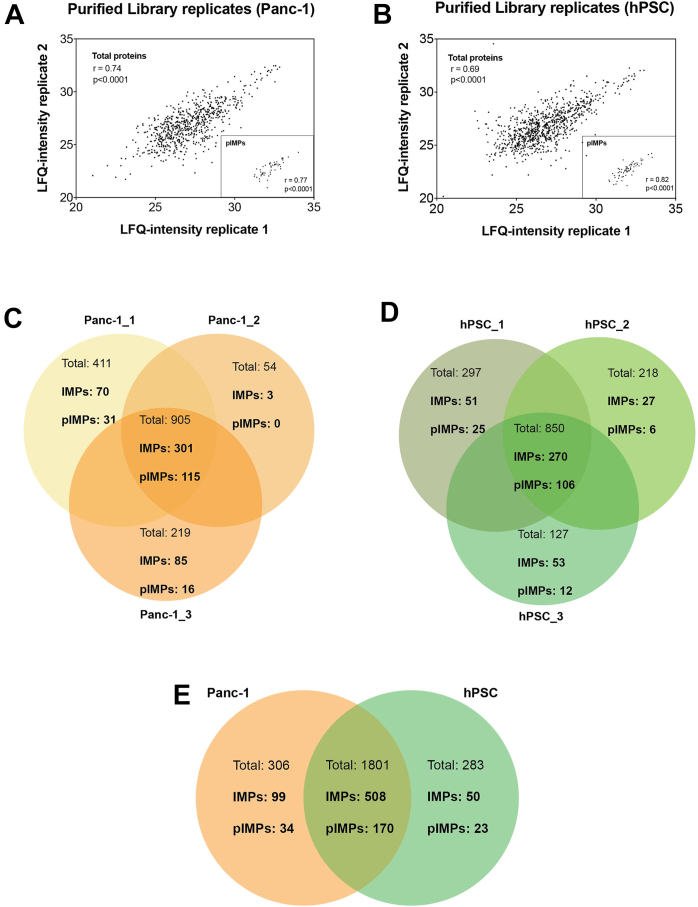
**Panc-1 and hPSC replicates.***A* and *B*, LFQ-analysis of the Purified Library prepared from Panc-1 and hPSC biological replicates. Three independent biological replicates were employed for each cell line. The Pearson correlation coefficient r was obtained using the MaxQuant and Perseus software. Note: Replicate 1 and 2 are presented here as a representation. The comparison between replicate 1 & 3, 2 & 3 is presented in the supplemental Fig. S3. *C*–*E*, protein ID overlap. Venn diagram representing the overlap across (*C*) biological replicates of Panc-1 Purified Libraries and (*D*) biological replicates of hPSC Purified Libraries. *E*, protein ID overlap between Panc-1 and hPSC Purified Libraries. LFQ, label-free quantitation.

**Fig. 5:**
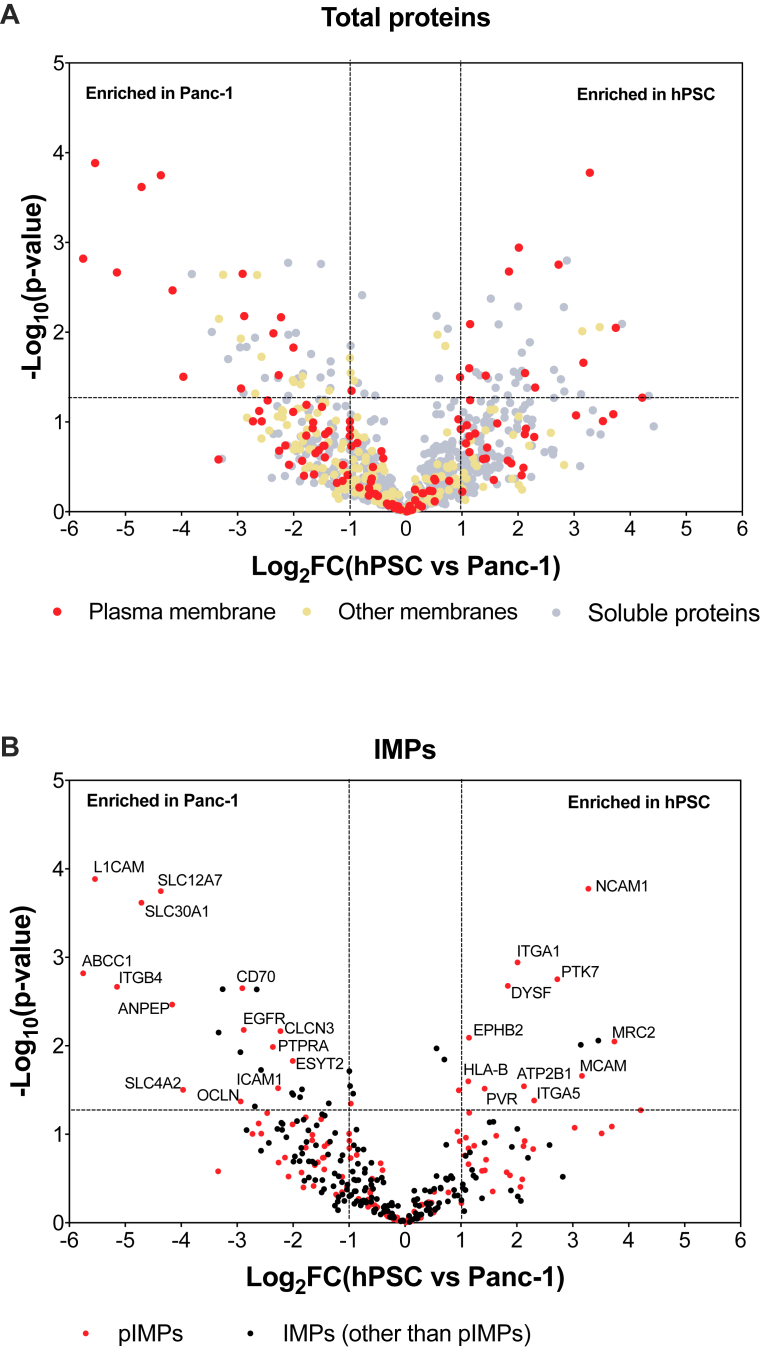
**Comparative analysis of the Panc-1 and hPSC Purified Libraries.** Volcano plot analysis of the (*A*) total proteins common to Panc-1 and hPSC. pIMPs are labeled in *red*, other IMPs are labeled in *light yellow*, and soluble proteins (without predicted TMS) are labeled in *gray*. *B*, IMPs common to Panc-1 and hPSC. pIMPs are labeled in *red*, and other IMPs are labeled in *black*. pIMPs with an absolute value of Log_2_FC ≥ 1 and *p* < 0.05 are considered differentially expressed across the two cell lines, and their gene names are labeled on the figure. FC, fold change; IMP, integral membrane protein; pIMP, plasma integral membrane protein; TMS, transmembrane segment.

Following the protocol established with the HeLa cells, the Panc-1 and the hPSC membrane proteomes were captured in His-tagged peptidiscs and affinity purified. The experiments were performed in biological triplicates (Figs. 4, S3 and supplemental File S4). In Panc-1, of the 162 pIMPs identified, 115 were present in all replicates. Of the 149 pIMPs identified in hPSC, 106 were present in all replicates (Fig. 4, *C* and *D*). Comparing Panc-1 and hPSC, 508 IMPs, including 170 pIMPs, were common in the two cell lines (Fig. 4*E*). The LFQ-intensity value for these common proteins was then used to estimate their relative abundance, and a student’s *t* test was conducted to determine the statistical significance of the observed protein differences (supplemental File S4). As a visual representation of data, we used a volcano plot (Fig. 5). We considered the proteins to be differentially expressed when the LFQ intensity FC is ≥2 (Log_2_FC ≥ 1) and the *p*-value <0.05 (-Log_10_(*p*-value) >1.3). The volcano plot also includes the protein subcellular location based on the GO-term annotation (Fig. 5*A*).

**Fig. 6:**
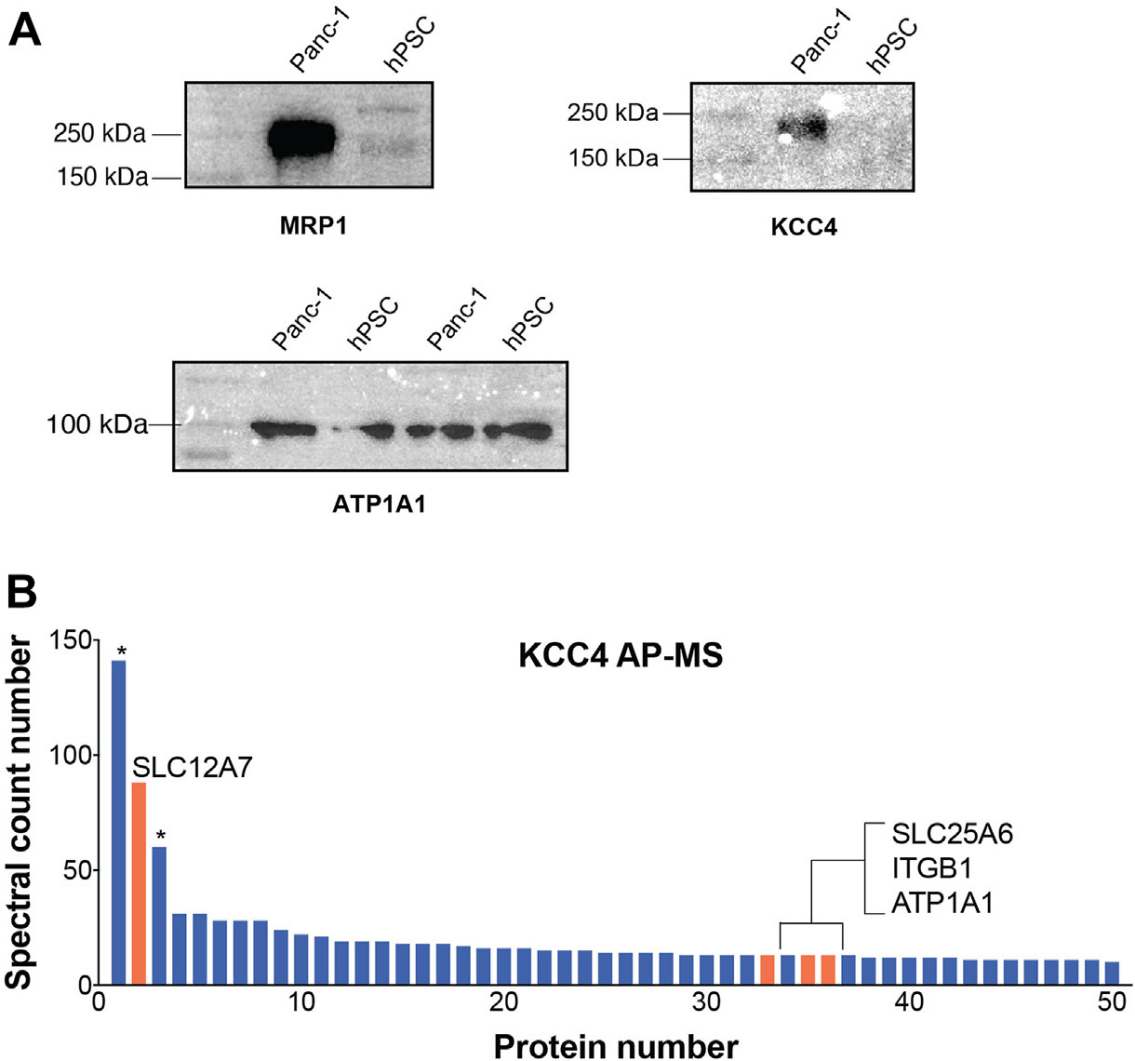
**Western blot analysis and KCC4 immunoprecipitation assay.***A*, Western Blot analysis of KCC4, MRP1, and ATP1A1 expression in Panc-1 and hPSC cells. The same amount (5 μg) of crude membranes from Panc-1 and hPSC cells were loaded onto 10% SDS-PAGE and analyzed by Western blot. The uncropped Western Blots and the corresponding silver-stained gel are shown in supplemental Fig. S5. *B*, immunoprecipitation of KCC4 (SLC12A7) from the Panc-1 peptidisc library and MS analysis of the resulting sample (AP/MS). The IMPs are colored *orange*, and the soluble proteins are colored *blue*. The (∗) correspond to the soluble proteins vimentin and heterogeneous nuclear ribonucleoproteins A2/B1, respectively. IMP, integral membrane protein; MS, mass spectrometry.

A total of 14 pIMPs were found enriched in the Panc-1 library relative to the hPSC library (Fig. 5*B* and Table 3). Encouragingly, transcriptomic data indicate that most of these proteins also have high expression levels in the Panc-1 cell line and, for the most part, are also overexpressed in the other 53 PDAC cell lines tested (supplemental Fig. S4). Specifically, L1CAM, ANPEP, ITGB4, and CD70 are considered specific biomarkers of the PDAC disease (55, 56, 57, 58, 59). The membrane transporter ABCC1 (MRP1), which confers multidrug resistance to cancer cells, was also enriched in the Panc-1 library, along with three other SLC transporters, SLC4A2, SLC30A1, and SLC12A7 (KCC4), plus the epidermal growth factor receptor EGFR and lipid transport protein ESYT2. The proteins MRP1 and KCC4 were selected for validation by Western Blot analysis (Fig. 6*A*). The Western Blot results corroborate nicely the MS analysis.

**Table 3:** Differentially expressed pIMPs identified in Panc-1 and hPSC Purified Libraries

Gene name	Fold change (Log_2_)	Literature evidence	Sample and method of the analysis
Enriched in Panc-1 over hPSC			
ABCC1	5.8 ± 1.2	Upregulated (82, 83)	PDA mouse model; RT-PCR
L1CAM	5.5 ± 0.8	Upregulated (82, 84)	Pancreas, Panc-1; IHC
ITGB4	5.1 ± 1.7	Upregulated (59)	Tissue, cell line; WB, IHC, RT-PCR
SLC30A1	4.7 ± 0.7	n.a.	
SLC12A7	4.4 ± 0.5	n.a.	
ANPEP	4.2 ± 1.3	Downregulated (56)	Bioinformatics; Gene Expression Omnibus
SLC4A2	4.0 ± 2.1	Upregulated (71)	Panc-1 and Panc1R; Proteomics
OCLN	2.9 ± 2.3	n.a.	
CD70	2.9 ± 0.8	Upregulated (58, 82, 85)	Panc-1; FACS, IHC, RT-PCR
EGFR	2.9 ± 0.8	Upregulated (86, 87)	Panc-1; WB, Database search
PTPRA	2.4 ± 1.2	Upregulated (88)	Bioinformatics; Gene Expression Omnibus
ICAM1	2.3 ± 0.9	Upregulated (89, 90)	Serum; ELISA
CLCN3	2.2 ± 0.7	n.a.	
ESYT2	2.0 ± 1.1	Upregulated (82, 91)	Biopsy samples; Onco-array
Enriched in hPSC over Panc-1			
MRC2	3.7 ± 1.6	Stromal signature gene (60, 61, 62)	Tissue; WB, expression array
NCAM1	3.3 ± 0.4	Increased expression (63)	Immortalized PSCs; RT-PCR
MCAM	3.2 ± 1.2	n.a.	
PTK7	2.7 ± 0.9	n.a.	
ITGA5	2.3 ± 0.4	Upregulated (64)	Tissue; microarray
ATP2B1	2.1 ± 1.4	Slightly higher in hPSC (66)	Panc-1 and hPSC; RT-qPCR
ITGA1	2.0 ± 0.2	n.a.	
DYSF	1.8 ± 0.5	n.a.	
PVR	1.4 ± 0.8	n.a.	
HLA-B	1.1 ± 0.8	n.a.	
EPHB2	1.1 ± 0.4	n.a.	

The average fold change (Log_2_) ± SD was calculated using LFQ intensity values across the triplicates in MaxQuant software. Column “Literature evidence” indicates that at least one previous publication has reported an upregulation of the listed pIMPs. The sample employed (cells, tissues, database) and the method of analysis in the corresponding publication is indicated. N.a.: no data available in the Panc-1 and hPSC cell lines.

In the hPSC library, 11 pIMPs were identified as enriched over the Panc-1 library (Fig. 5*B* and Table 3). Proteins such as MRC2, a signature protein in pancreatic stroma (60, 61, 62), or ITGA5, NCAM1, and ATP2B1 have been reported earlier to have high expression in the hPSC cell line (63, 64, 65). The other seven pIMPs we have identified have not been documented in previous studies. Their potential role in pancreatic stellate cell biology in pancreatic cancer development may be worth exploring in future studies.

### Immunoprecipitation of SLC12A7 from the Panc-1 Peptidisc Library

We next examined the usability of the peptidisc library. Multi-TMS proteins, such as GPCRs and SLC transporters, are valuable targets for biomedical discovery (66, 67). However, their hydrophobicity renders purification and *in vitro* characterization difficult. Since the peptidisc stabilizes MPs in a detergent-free water-soluble state, we tested whether proteins in the library, such as SLC12A7 (KCC4), can be isolated. The Panc-1 membrane proteome was trapped in peptidiscs, followed by incubation with an anti-KCC4 antibody. The complex was captured on a Protein A affinity resin and washed twice to remove unspecific proteins. Finally, the eluted sample was trypsin digested and analyzed by LC-MS/MS. A total of 399 proteins were identified with at least two spectral counts (supplemental File S5). For visualization, we plotted the spectral count number for the top 50 proteins identified (Fig. 6*B*). From the plot, it is evident that KCC4 (SLC12A7) has been isolated using the anti-KCC4 antibody.

## Discussion

This study extends the application of the peptidisc to the mammalian membrane proteome to identify and isolate proteins that are integral to the membrane because of an α-helical TMS. This class of protein is of great pharmaceutical interest as it includes most cell markers and disease biomarkers, which, given their cell surface exposure, can be targeted with drugs and biologics. The precise characterization of the membrane proteome, especially at the cell surface, remains complicated, however, because this proteome is inherently insoluble and in low abundance. Consequently, it is harder to isolate and vastly underrepresented in the proteomic datasets (46, 68, 69). To help circumvent the limitations, we employed the His-tagged peptidisc to purify the cell membrane proteome *via* Ni-NTA chromatography.

We used the HeLa cell line as a reference. Membranes were isolated by ultracentrifugation and solubilized with a mild detergent, and the extracted proteins were trapped and purified in His-tagged peptidiscs. This simple workflow—no extensive fractionation and limited MS analysis (60 min)—produced a library containing ∼500 different IMPs, with half predicted to be located in the plasma membrane. The other half comprised proteins from smaller organelles, such as ER, Golgi, and cell trafficking vesicles, probably due to similar densities and membrane contact sites leading to co-isolation during ultracentrifugation. Notably, the library annotation did not reveal an apparent bias towards protein function, as the general protein categories expected for the HeLa cell were all present (Table 2). There was also no evident bias towards the isolated protein size, as the library contained proteins having 1 to 17 TMS and a molecular weight ranging from 4 to ∼800 kDa (supplemental File S1).

The method’s sensitivity was assessed by examining the abundance of the pIMPs over total proteins within and across the samples (Figs. 1 and 2). As expected, MP detection was significantly improved upon library purification. In the final library, around twice more pIMPs were present within the top 400 proteins isolated (Fig. 2). The pIMPs enrichment was also evident when comparing our dataset to the Protein Abundance Database (PAXdb) (https://www.pax-db.org). Some ABC transporters (ABCC2, ABCC3), SLC transporters (SLC7A2, SLC26A6, SLC39A8), as well as GPCRs (OR10P1, RXFP1) and adhesion molecule (EPCAM), all reported at low abundance in the PAXdb database (bottom 50%), were ranked within the top 25% in our list. Additionally, of the 249 pIMPs in our dataset, *∼*15% (37 IDs) were either completely absent or located in the bottom 5%-10% in the PAXdb database. Clearly, removing the “spectral space” occupied by soluble contaminants is essential to augment the MS detection sensitivity for MPs. Accordingly, a recent study also reports that washing the membrane fraction with chaotropic agents significantly increases pIMPs’ detection sensitivity (69).

We then applied our method to compare the membrane proteomes of two human pancreatic cell lines, Panc-1 and hPSC. We identified 25 cell surface MPs differentially expressed across the two cell lines (FC ≥2 and *p* < 0.05). Of the 14 pIMPs upregulated in Panc-1, four pancreatic cancer biomarkers were previously reported (ANPEP, L1CAM, ITGB4, and CD70; Table 3 and reference within). We also identified MRP1 (ABCC1), a protein responsible for chemoresistance and typically upregulated in cancer cells (70), and ITGB4, which has a key role in tumorigenesis (59). Three SLC transporters (SLC4A2, SLC30A1, and SLC12A7) were upregulated. The anion exchange protein 2 (SLC4A2) was reported upregulated in a gemcitabine-resistant Panc-1 cell line at the protein levels (71). The SLC12A7 (or KCC4) has a potassium/chloride symporter activity, and systematic gene amplification was observed in adrenocortical carcinoma, where it promotes higher cell motility and invasiveness (72, 73, 74). For SLC30A1, a proton-coupled zinc antiporter, its upregulation is found in gastric cancer and cervical carcinoma (75, 76). Thus, the potential role of these three SLCs in PDAC occurrence is a promising avenue for future exploration. Of the 11 pIMPs enriched in the hPSC library, some were previously identified as stellate cell markers (*i.e.*, MRC2, NCAM1, and ITGA5; Table 3 and reference within), but most others have not yet been studied in the context of PDAC progression. Collectively, these results validate the peptidisc as a method to survey and compare the cell plasma membrane proteomes. Such comparison is critically important for the discovery of tumor-specific cell surface markers (26) or for developing effective targeted immunotherapy, such as chimeric antigen receptor T cell immunotherapies or monitoring cell surface changes after, for example, drug treatment.

From a technical point of view, we see several other advantages of implementing a peptidisc-based approach for eukaryotic cell analysis. The survey of the pIMP proteome with peptidisc does not depend on surface labeling ((23, 24) for a review of other methods), and the construction of the peptidisc library does not require living cells. The same workflow can also be applied to characterize the membrane proteome in intact tissues or organs, where other methods are limited by the penetration of the labeling reagents (77). Finally, a detergent removal step is not necessary, thus reducing the risk of peptide loss (24, 26). Several methods exist to remove detergents before MS analysis and thereby increase detection efficiency, including filter-aided sample preparation, S-Trap, SP3, and recently SP4 (78, 79, 80, 81). Still, in all cases, MPs are irreversibly denatured with detergents or organic solvents, whereas in peptidisc, MPs are preserved in a water-soluble state, which is a distinctive advantage for downstream analysis, such as protein purification, binding assays, and screening of libraries of antibodies or small molecules.

Further development to better implement the peptidisc in the proteomic analysis is possible, but some inherent limitations exist. One is the amount of cell material necessary to construct the library (∼20–40 million cells), which renders the method with limited biological material. Secondly, multiple copies of the peptidisc peptide are necessary to keep MP water-soluble. We estimated that 10 to 12 peptidisc peptides are bound to a multi-TMS protein (35). Although peptidisc peptides are compatible with trypsin digestion and MS analysis, this large amount relative to total proteins can lead to MS signal suppression. Further peptide fractionation or data-independent acquisition might be needed to address this issue.

## Data Availability

The mass spectrometry proteomics data have been deposited to the ProteomeXchange Consortium *via* the PRIDE [1] partner repository with the dataset identifier PXD039990 and PXD041913. For annotated spectra, the data have been uploaded to MS-viewer with the search keys “kefaqk11ru” and “msekhytirl”.

## Supplemental data

This article contains supplemental data. The “supplemental Table S2_experimental group #1” in the reference study (46) and “supplemental File S1” in the reference study (47) were used to compare with and benchmark our study (see Table 1 and supplemental Fig. S1).

## Conflict of interest

Franck Duong is the scientific founder of Peptidisc Biotech. All other authors declare no competing interests.

## Figures and Tables

**Figure S2A:**
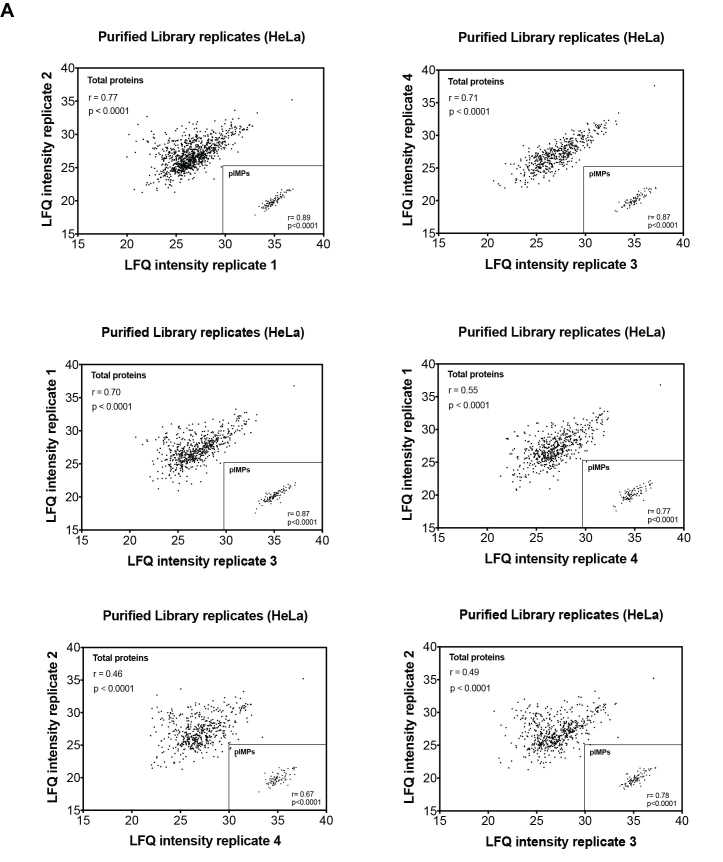


**Figure S2BC:**
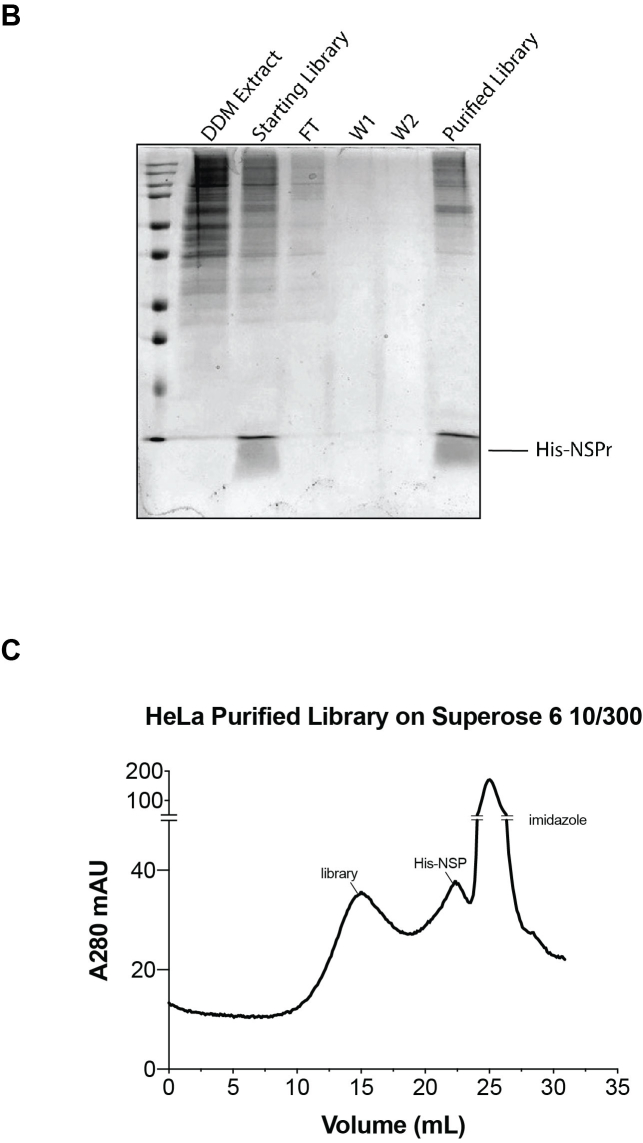


**Figure S5:**