# Supplementary material for: A Peptidisc-Based Survey of the Plasma Membrane Proteome of a Mammalian Cell
Source: Mol Cell Proteomics. 2023 Jun 7;22(8):100588. doi: 10.1016/j.mcpro.2023.100588 (PMC10416069; doi:10.1016/j.mcpro.2023.100588)
Supplement: Supplemental data [file mmc1.docx]

**A Peptidisc-based Approach to Survey the Plasma Membrane Proteome of a Mammalian Cell**

Zhiyu Zhao, Arshdeep Khurana, Frank Antony, John W. Young, Keeley G. Hewton, Zora Brough, Tianshuang Zhong, Seth J. Parker, and Franck Duong van Hoa

**Supplementary data**

**Figure S1.** The comparison of IMPs and pIMPs across three methods.

**Figure S2**. HeLa Purified Library.

**Figure S3**. LFQ-analysis of the Purified Library prepared from Panc-1 and hPSC biological replicates.

**Figure S4**. Transcriptomic data for the pIMPs found enriched in Panc-1.

**Figure S5**. Uncropped Western Blot membrane image (related to Figure 6A) and the corresponding silver-stained SDS-PAGE.

**Supplementary figures**

**
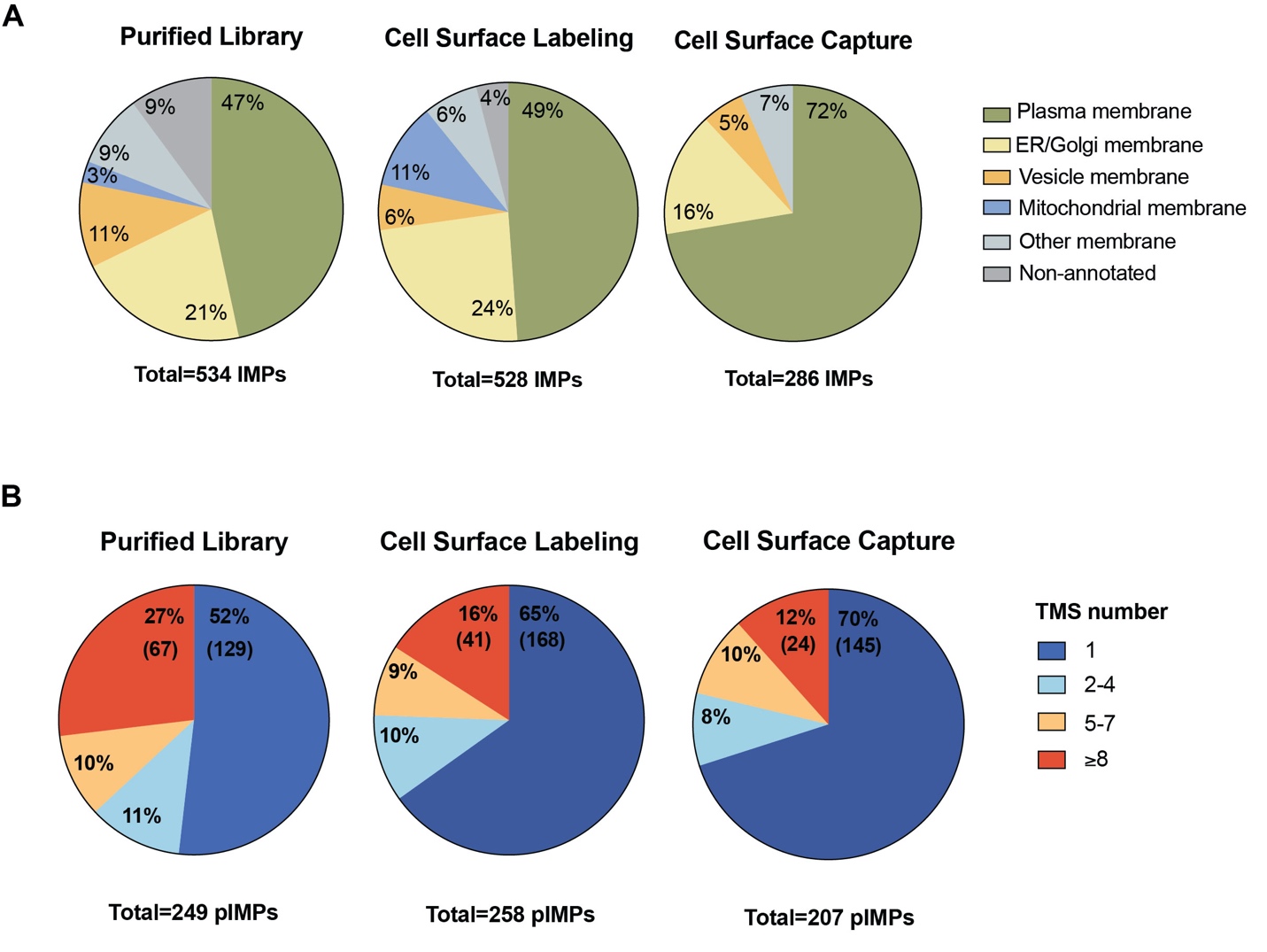
**

**Figure S1.** The comparison of IMPs and pIMPs across three methods. (**A**) Subcellular location comparison of identified IMPs in each method. The IMPs are predicted using Phobius web server. The proteins are then analyzed using Go-term “Subcellular Location [CC]” and “Gene Ontology (cellular component)” in UniprotKB. (**B**) The comparison of TMS number of pIMPs. The TMS number of each pIMP was predicted using Phobius

**
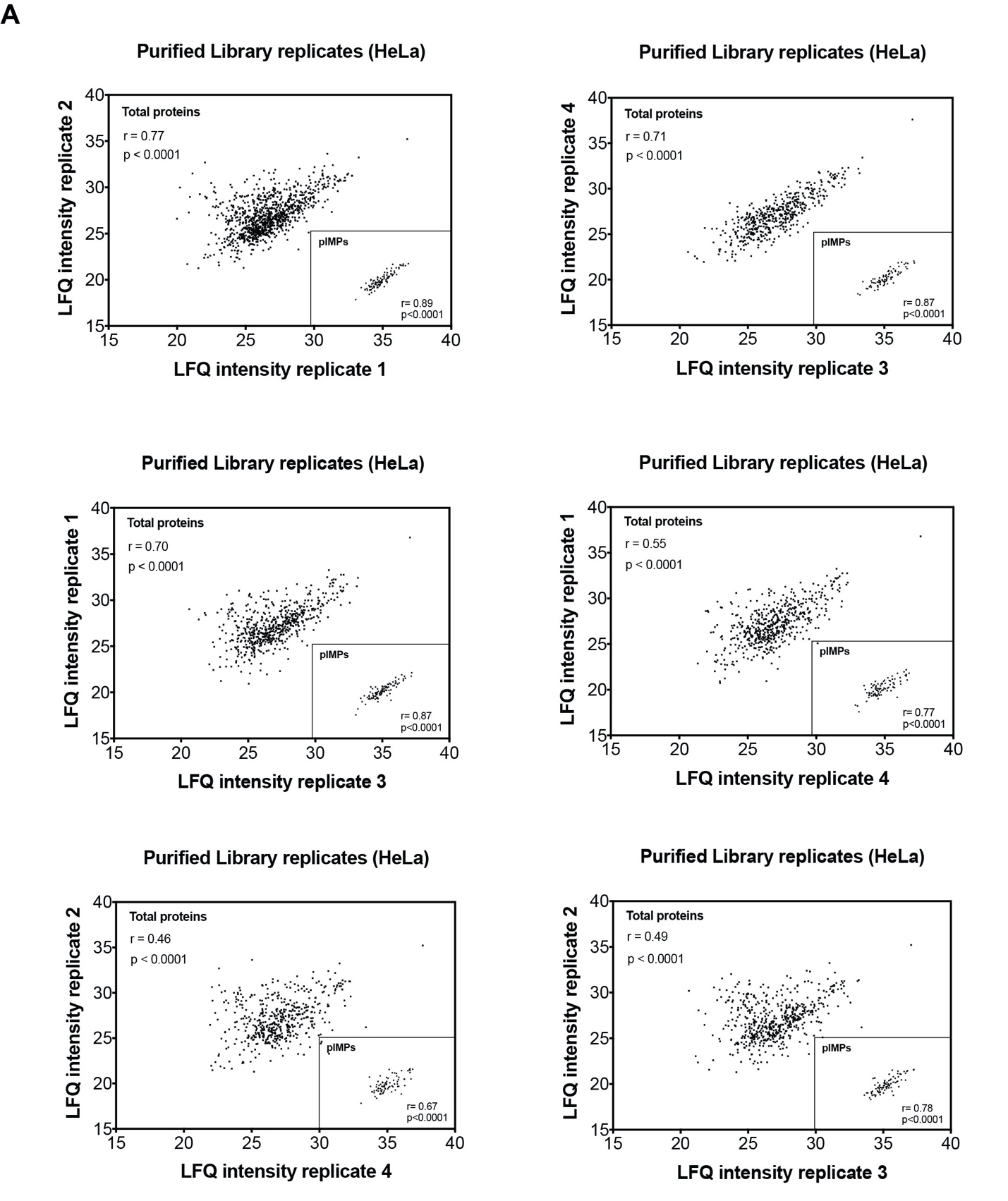
**

**
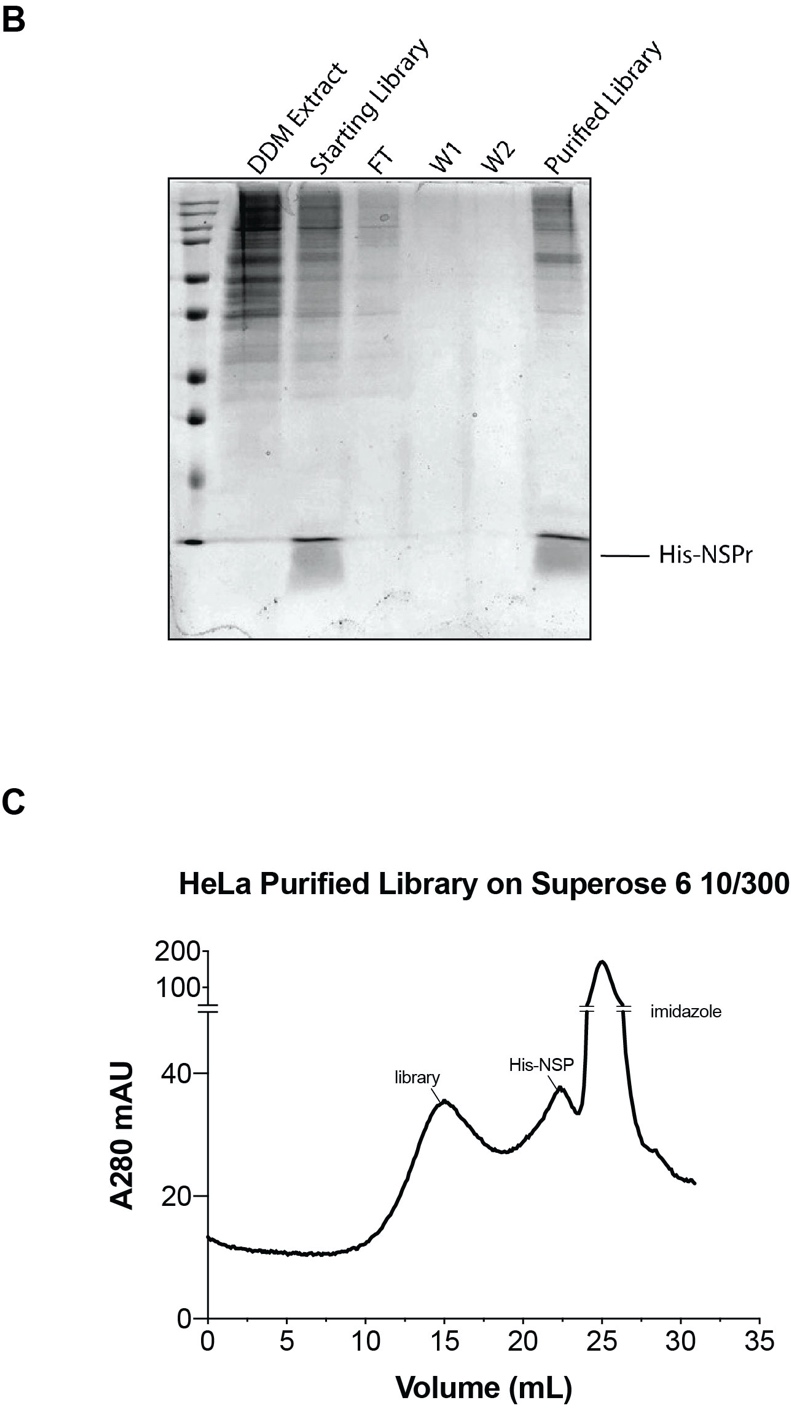
**

**Figure S2**. HeLa Purified Library. **(A)** The LFQ-analysis was performed on quadruple biological replicates of the HeLa cell Purified Library. The Pearson correlation coefficient r was obtained using the MaxQuant and Perseus software. **(B)** The protein content of the DDM Extract, Starting Library, and Purified Library was analyzed on SDS-PAGE followed by Coomassie Blue staining prior to protein digestion. **(C)** Gel filtration chromatogram of HeLa Purified Library on column Superose 6 10/300. 400 µg of the purified library (in 500 µL) was injected into the column.

**Figure S3**. LFQ-analysis of the Purified Library prepared from Panc-1 and hPSC biological replicates. The comparison between biological replicate 1 and 3, 2 and 3 is measured using Pearson correlation coefficient r.

**Figure S4**. Transcriptomic data for the pIMPs found enriched in Panc-1. The plot represents the expected expression across a lineage of 53 different pancreatic cancer cell lines. The location of the Panc-1 cell line within the pancreatic lineage is indicated by (*). These transcriptomic data were obtained from DepMap (<https://depmap.org/portal/>). The dashed line represents an arbitrary cutoff, which sets Log_2_(TPM+1) to 2.

**
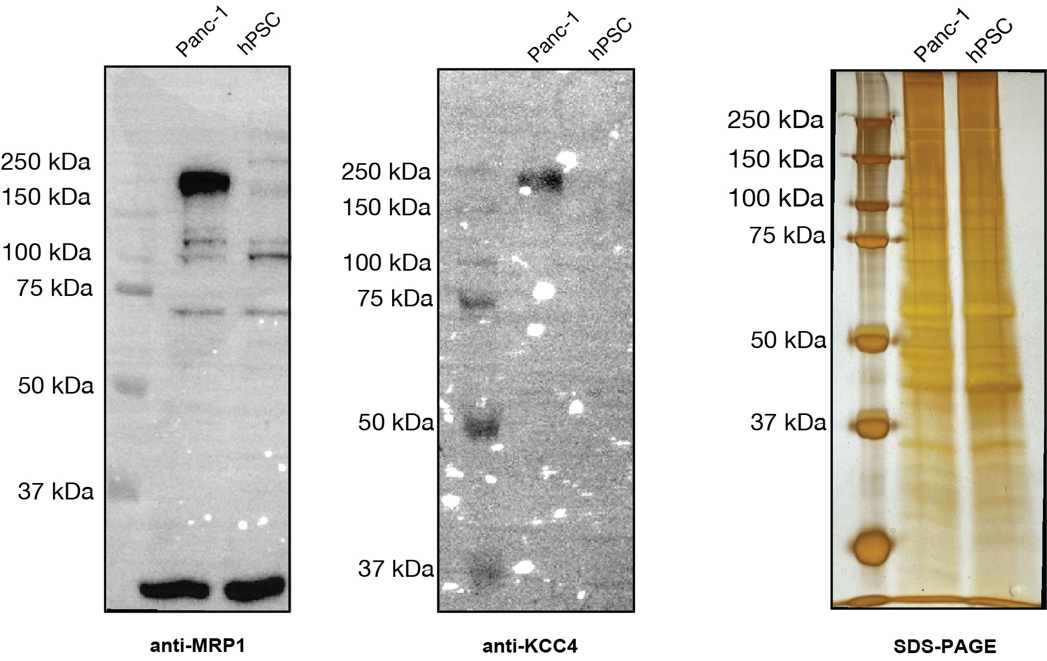
**

**Figure S5**. Uncropped Western Blot membrane image (related to Figure 6A) and the corresponding silver-stained SDS-PAGE.
