# Supplementary figures and images for: A Peptidisc-Based Survey of the Plasma Membrane Proteome of a Mammalian Cell
Source: Mol Cell Proteomics. 2023 Jun 7;22(8):100588. doi: 10.1016/j.mcpro.2023.100588 (PMC10416069; doi:10.1016/j.mcpro.2023.100588)

**A**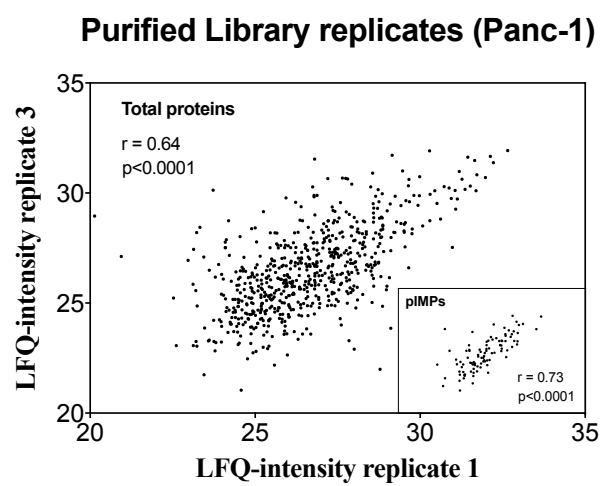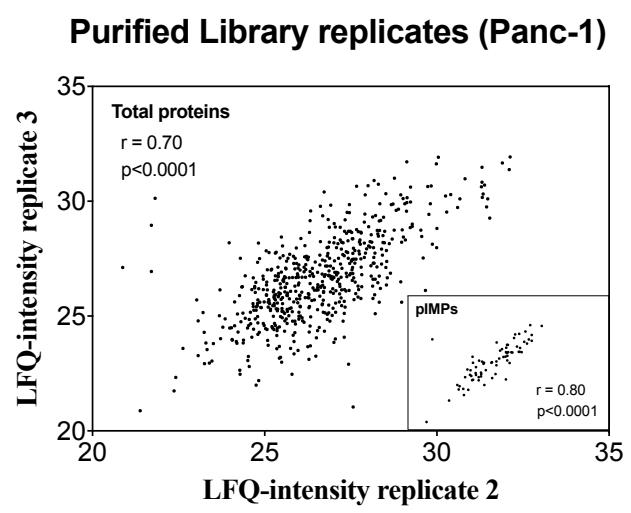**B**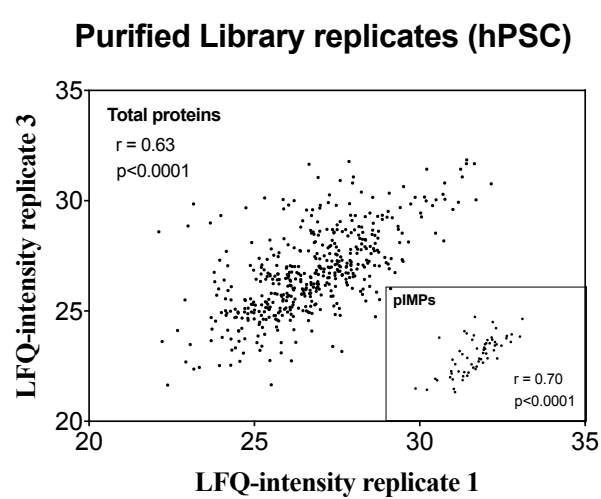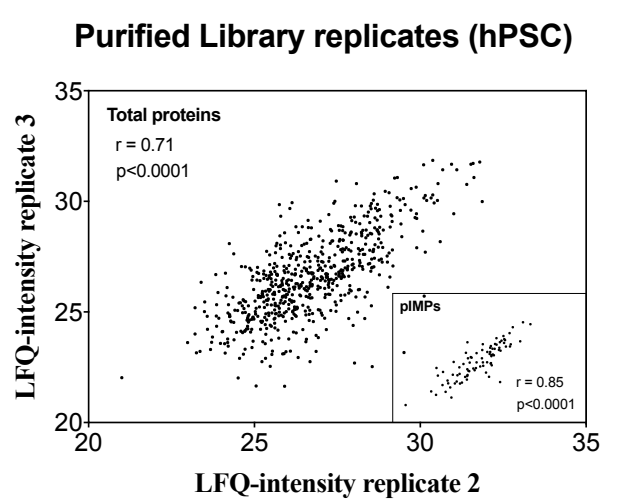

Supplement: Figure S3 [file mmc8.pdf]

# PDAC Transcriptomics DepMap

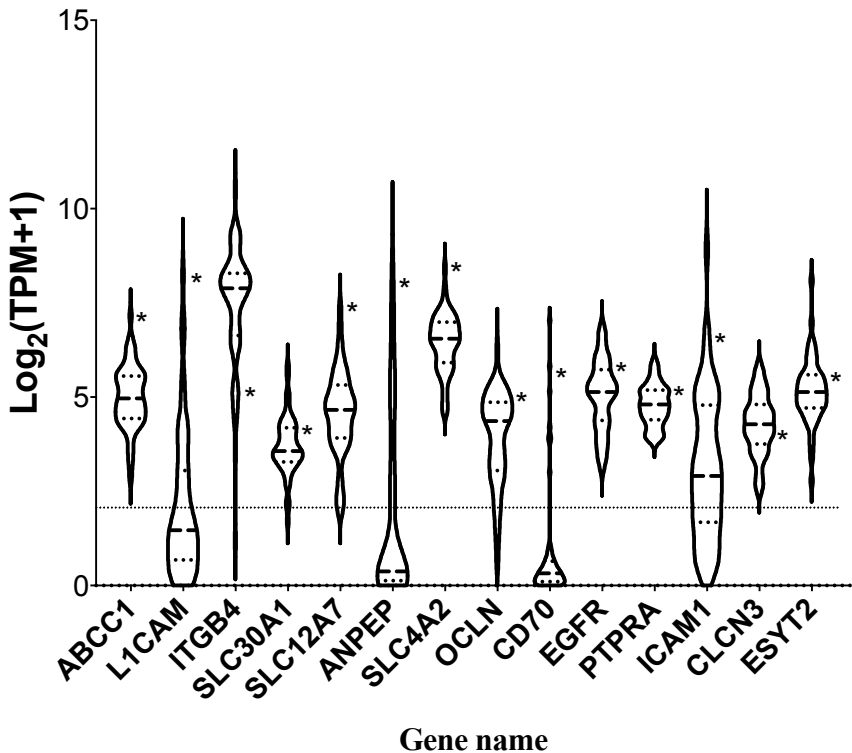

Supplement: Figure S4 [file mmc9.pdf]
